# Evaluation of left atrial volume and function using single-beat real-time three-dimensional echocardiography in atrial fibrillation patients

**DOI:** 10.1186/s12880-017-0215-7

**Published:** 2017-07-21

**Authors:** Qian Zhang, Ju-fang Wang, Qing-qing Dong, Qing Yan, Xiang-hong Luo, Xue-ying Wu, Jian Liu, Ya-ping Sun

**Affiliations:** Department of Echocardiography, Shanghai General Hospital, Shanghai Jiao Tong University School of Medicine, No.100 Hai Ning Road, Shanghai, 200080 China

**Keywords:** Real-time three-dimensional echocardiography, Atrial fibrillation, Left atrial volume, Left atrial function

## Abstract

**Background:**

This study was aimed to evaluate the feasibility and accuracy of real-time three-dimensional echocardiography (RT-3DE) measurement of left atrial (LA) volume and function in comparison with two-dimensional echocardiography (2DE) measurements in atrial fibrillation (AF) patients.

**Methods:**

A total of 50 pairs of AF patients and healthy controls were enrolled in this study. Indexed LA end-diastole volume (ILAEDV) and indexed LA end-systolic volume (ILAESV), as well as LA function indices such as segmental LA ejection fraction (LAEF), were assessed using 2DE Simpson’s method and the RT-3DE method.

**Results:**

The images showed that regional LA volume–time curves and LAEF were disordered in AF patients. ILAEDV and ILAESV were markedly increased and global LAEF was significantly decreased in AF patients compared with those in healthy controls (*P* < 0.01). No significant differences were found in ILAEDV, ILAESV, and LAEF levels as determined by the RT-3DE method or 2DE Simpson’s method. Bland–Altman analysis showed that the two methods agreed well for measuring ILAEDV, ILAESV, and segmental LAEF.

**Conclusion:**

The RT-3DE method may be a feasible and accurate method for evaluating LA volume and function of AF patients in clinical practice.

**Electronic supplementary material:**

The online version of this article (doi:10.1186/s12880-017-0215-7) contains supplementary material, which is available to authorized users.

## Background

Atrial fibrillation (AF) is one of the most common sustained cardiac arrhythmias and is associated with increased morbidity and mortality, with its incidence ranging from 2.73% in 1993 to 2.83% in 2007 [1, 2]. There is strong evidence that left atrial (LA) volume and ejection fraction (EF) are important factors that influence AF development [3, 4]. Previous studies indicated that measuring LA volume is valuable for predicting AF recurrence [5, 6]. Segmental EF is a more sensitive index of segmental LV function than global EF in AF patients [7].

Two-dimensional echocardiography (2DE) using Simpson’s method has been widely used for assessing LA volume and segmental LAEF in previous studies [8, 9]. However, this method not only requires the acquisition of a series of contiguous images that cover the entire LA but also requires manual tracking, making it time consuming. Moreover, geometric assumptions that have a risk for underestimating volumes are required for the 2DE method [10, 11]. Three-dimensional echocardiography (3DE) is a recently developed technology that has an advantage in overcoming the geometric limitations of 2DE [12]. The single-beat real-time 3DE (RT-3DE) method has been widely used to produce integrated, instantaneous, and large volumes at high volume rates of 3D cardiac images in a single cardiac cycle and to automatically measure the LA volume [13]. RT-3DE may be feasible for clinical application, superior to 2DE for patients with severe mitral regurgitation [14] and aortic regurgitation [15]. However, few studies have focused on the clinical use of RT-3DE for detecting the LA volume and EF, compared with 2DE.

In this study, segmental LAEF and LA volume in AF patients and healthy controls were measured using the single-beat RT-3DE method and 2DE Simpson’s method. The study assessed the accuracy and feasibility of RT-3DE measurements of LA volume and function compared with standard 2DE measurements in AF patients.

## Methods

### Subjects

Between June and September 2014, a total of 50 consecutive AF patients who visited the hospital for radiofrequency ablation were enrolled. AF was diagnosed by combined conventional electrocardiography (ECG) and dynamic electrocardiography (DCG). The conventional 12-lead ECG was used for recording the rate and rhythm of heartbeats in the patients in a resting condition, and DCG monitored the electrical activity in the states of resting, activity, working, studying and sleeping for consecutive 24 h. If AF could not be confirmed by ECG, patients were subjected to DCG monitor from 9 o’clock to 9 o’clock on the next day. Patients with other cardiovascular diseases, metabolic disorders, anemia, liver and renal failures, and lung diseases that could affect cardiac morphology and function were excluded. A total of 50 age- and sex-matched healthy volunteers who underwent a routine physical examination at our hospital were included as the control group.

### 2DE Simpson’s measurement

Participants were placed in the left lateral position and connected to an electrocardiogram monitor. The apical four-chamber view was obtained using a 4V1c transducer (1–4 MHz). The frame rate was 51 ± 5 frames/s. Segmental LAEF, LA end-diastolic volume (LAEDV), and LA end-systolic volume (LAESV) were measured using a Simpson’s method from the apical four-chamber view [16, 17]. The measurement was performed using an echocardiographic system (Siemens Acuson SC2000 diasonograph, Siemens Medical Solutions USA). LAEDV and LAESV were adjusted for body surface area (BSA) and were defined as indexed LA end-diastolic volume (ILAEDV) and indexed LA end-systolic volume (ILAESV).

### RT-3DE measurement

To conduct the apical four-chamber view, the 4Z1c probe (1–4 MHz) was used to collect LA images during a breath-hold over 5 s. The detectable depth was 16 cm, and the 3D pyramid scanning was 90° × 90° with temporal resolution of ≥40 frames/s. Meanwhile, the plane images of the apical four-chamber, three-chamber, and short-axis views were synchronously displayed (Additional file 1: Fig. S1). The full image for LA volume (Additional file 2: Fig. S2) was analyzed by manual tracking of LAED and LAES endocardium (Additional file 3: Fig. S3). Data of segmental LAEF, LAEDV, and LAESV were obtained. LAEDV and LAESV were corrected for BSA and recorded as ILAEDV and ILAESV. Regional LA volume–time curves and segmental LAEF were constructed using segmental values representing a whole cardiac cycle.

### Interobserver variability

All the above analyses were performed by an experienced physician who was blinded to the group assignment and subjects’ clinical status. To determine the interobserver variability, all measurements were repeated by a second physician blinded to the values obtained by the first physician, after an average of 1 week. The 2DE Simpson’s measurement and RT-3DE measurement were completed within 2 h of each other in one patient. Patients and healthy controls were evaluated in the same examination room. AF patients were examined and evaluated before radiofrequency ablation.

### Statistical analysis

All data are presented as mean ± standard deviation (SD). Paired *t* test was used to compare continuous variables between the control and case groups. Bland–Altman analysis was used to assess the agreement between 2DE Simpson’s measurement and RT-3DE measurement. The agreement between the two methods (2DE Simpson’s and RT-3DE) was expressed as 95% limits of agreement, as recommended by Bland and Altman [18]. All analyses were performed using the Stata software (version 11.2; StataCorp, TX, USA). *P* values of <0.05 were considered to be significant.

## Results

### Characteristics of the subjects

There were 29 male and 21 female subjects in the control group (mean age, 60.36 ± 1.30 years) and 32 males and 18 females in the case group (mean age, 60.06 ± 1.12 years). BSA was 1.61 ± 0.016 m^2^ and 1.63 ± 0.017 m^2^ for the control and case groups, respectively. No significant differences were observed with regard to age (*P* = 0.22), sex (*P* = 0.54), and BSA (*P* = 0.11) of the subjects in the two groups.

### RT-3DE images of LA volume, volume variations, and segmental LAEF during a single heartbeat

**Fig. 1:**
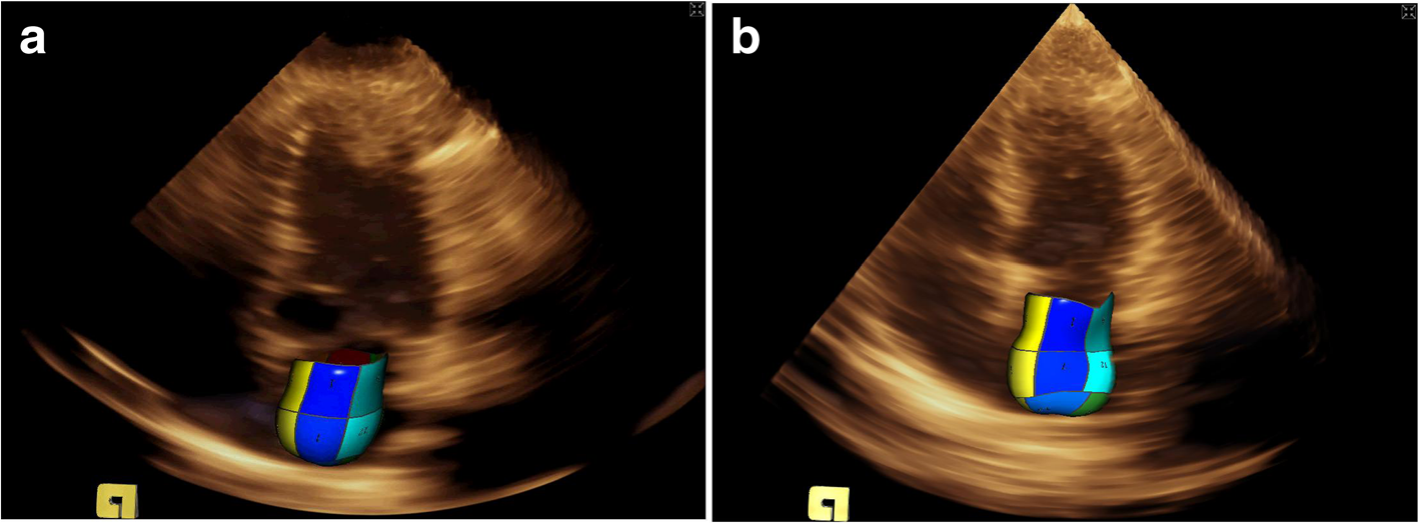
Real-time three-dimensional echocardiography (RT-3DE) images of left atrial volume of healthy controls (**a**) and patient cases (**b**)

**Fig. 2:**
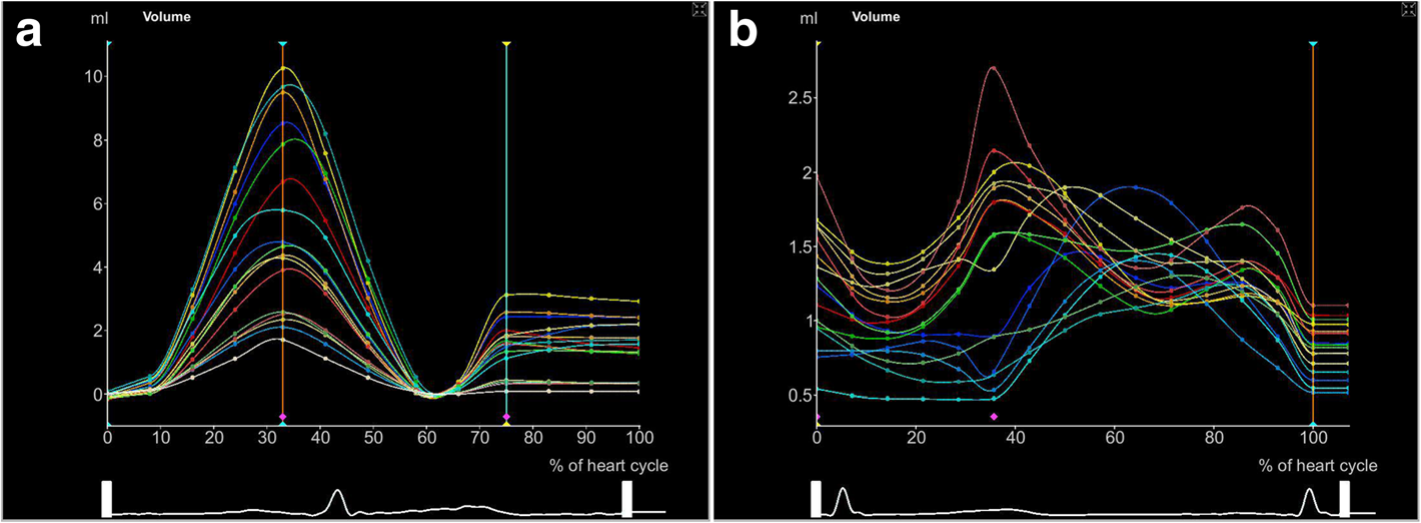
The regional left ventricular volume–time curve of healthy controls (**a**) and patient cases (**b**) by real-time three-dimensional echocardiography (RT-3DE)

**Fig. 3:**
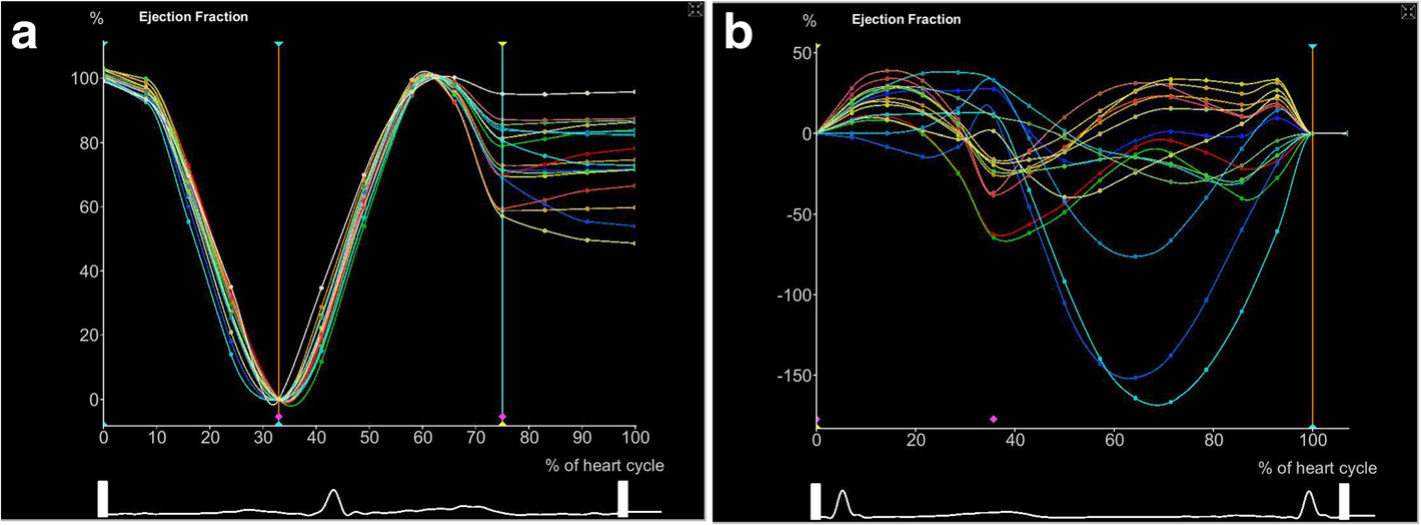
Segmental ejection fraction (EF) (%) of healthy controls (**a**) and patient cases (**b**) by real-time three-dimensional echocardiography (RT-3DE)

Compared with healthy controls, BSA-adjusted LA volume was greater in age-, sex- and BSA-matched AF patients (Fig. 1). Regional LA volume–time curves (Fig. 2a) and segmental LAEF during the cardiac cycle (Fig. 3a) of the control group were orderly arranged; in contrast, the curves in the case group were unordered (Fig. 2b and Fig. 3b).

### Comparison of LA volume and segmental LAEF between 2DE biplane Simpson’s measurement and RT-3DE measurement

The RT-3DE measurement showed that ILAEDV and ILAESV levels were significantly increased in the case group compared with the control group, but LAEF levels significantly decreased (all *P* < 0.01) (Table 1).

**Table 1:** Comparison of ILAEDV, ILAESV and segmental LAEF between two groups

Parameter	Method	Control	Case	*P* value
ILAEDV (ml)	Simpson’s	12.60 ± 0.35 (0.20)	27.40 ± 1.50^a^(0.39)	<0.01
	RT-3DE	12.56 ± 0.35 (0.20)	27.38 ± 1.51^a^(0.39)	<0.01
ILAESV (ml)	Simpson’s	8.40 ± 0.25 (0.21)	22.26 ± 1.17^a^(0.37)	<0.01
	RT-3DE	8.41 ± 0.25 (0.21)	22.28 ± 1.17^a^(0.37)	<0.01
Segmental LAEF (%)	Simpson’s	46.94 ± 0.92 (0.14)	21.87 ± 1.20^a^(0.40)	<0.01
	RT-3DE	47.49 ± 0.76 (0.11)	21.52 ± 1.30^a^(0.43)	<0.01

Data were presented as mean ± standard error of mean (coefficient of variation). *ILAEDV* indexed left atrial end-diastolic volume, *ILAESV* indexed left atrial end-systolic volume, *LAEF* left atrial ejection fraction. ^a^, significant difference between the case group and control group

Similarly, the results of 2DE Simpson’s measurement showed that ILAEDV and ILAESV levels were markedly increased in the case group compared with the control group (all *P* < 0.01). Moreover, the case group had lower global LAEF than the control group (*P* < 0.01) (Table 1).

However, no significant differences were found in ILAEDV (*P* = 0.57 in control, *P* = 0.066 in case), ILAESV (*P* = 0.84 in control, *P* = 0.93 in case), and LAEF (*P* = 0.062 in control, *P* = 0.17 in case) levels between the RT-3DE measurement and 2DE Simpson’s measurement.

### Bias between 2DE Simpson’s measurement and RT-3DE measurement of LA volume and segmental LAEF

**Fig. 4:**
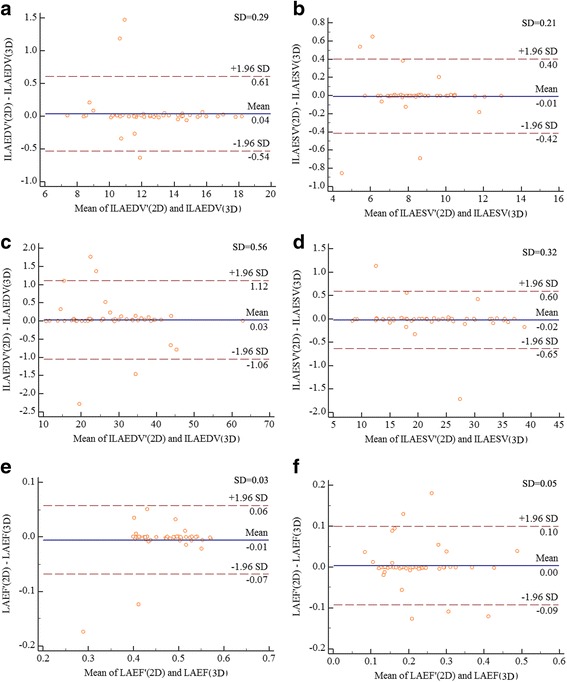
**a** The correlation of 2DE and RT-3DE measurement of ILAEDV in control group; **b** the correlation of 2DE and RT-3DE measurement of ILAESV in control group; **c** the correlation of 2DE and RT-3DE measurement of ILAEDV in case group; **d** the correlation of 2DE and RT-3DE measurement of ILAESV in case group; **e** the correlation of 2DE and RT-3DE measurement of IAEF in control group; **f** the correlation of 2DE and RT-3DE measurement of IAEF in case group

The Bland–Altman plots (Fig. 4) showed a good agreement between 2DE biplane Simpson’s measurement and RT-3DE measurement. The Bland–Altman plots graphically showed that the mean differences (between 0.02 and 0.04) were nearly 0 for all tests. No systematic differences in SD (between 0.03 and 0.56) and random error (95% limits of agreement) were found.

## Discussion

There were no significant differences in LAEDV, LAESV, and segmental LAEF between the results of RT-3DE measurement and 2DE Simpson’s measurement. Furthermore, the two methods agreed well for measuring segmental LAEF. The study results suggested that single-beat RT-3DE measurement is as accurate and feasible as 2DE Simpson’s measurement of LA volume and function in AF patients.

Evidence has proved that 2DE Simpson’s measurement is reliable for assessing LA volume and function [10]. The 2DE method requires the measurement of the apical four-chamber view and apical two-chamber view to evaluate LA volume and function, whereas the RT-3DE method only requires the measurement of the apical four-chamber view for the acquisition of related data. The 2DE Simpson’s method is more complicated and could be more time consuming than the RT-3DE method. The single-beat RT-3DE method has overcome the drawbacks of 2DE in geometric assumption and offers 3D pyramid scanning of 90° × 90° and a 16-cm depth, which covers the entire LA volume with acceptable image quality and eliminates the stitch artifact of previous RT-3DE [19]. The single-beat RT-3DE method can accurately and rapidly calculate the LA volume and function index and provide a visual observation of LA volume and function at each stage of every cardiac cycle. These superior features will be beneficial for AF patients with arrhythmia.

Previous studies have validated the feasibility of RT-3DE in clinical assessment. RT-3DE was used by Cong et al. to determine LA volume and function during normotensive and preeclamptic pregnancy [20]. Although Marsan et al. [21] measured LA volumes and segmental LAEF using RT-3DE in AF patients, LA volume variations in a cardiac cycle were not visually presented as images, and the comparison between new RT-3DE and conventional 2DE was not performed. Furthermore, there were no significant differences in ILAEDV, ILAESV, and segmental LAEF levels that were determined using the single-beat RT-3DE method and 2DE Simpson’s method. Moreover, the two methods had good agreement for measuring segmental LAEF. Although the high correlation between LA volumes and LAEF using 2DE and RT-3DE may have been because of the single physician performing the measurements to some extent, these findings still suggested that the RT-3DE method was as feasible as the 2DE method for assessing LA volume and function.

In addition, LA volume and segmental LAEF are suggested to be robust markers of adverse cardiovascular events [22] and are found to reflect LA dysfunction [23, 24] and are associated with the risk for incident AF [4, 25, 26]. LA volume has a predictive role for congestive heart failure in AF patients [24]. In our study, regional LA volume–time curves of AF patients were discordant with those of healthy controls, and LA volume-related ILAEDV and ILAESV levels were remarkably increased. In addition, segmental LAEF was clearly reduced in AF patients compared with that in healthy controls. These results suggested LA dysfunction and unsynchronized LA volume enlargement in a cardiac cycle in AF patients, which were largely consistent with previous studies [27–29].

This study is limited by the fact that the population size is relatively small. In addition, LA function of AF patients was preliminarily evaluated without long-term follow-up data after radiofrequency ablation. Therefore, an investigation of a large number of AF patients with a long-term follow-up is required for confirming the results of this study. Moreover, the effects of 2DE and RT-3DE measurements were not compared with a gold standard such as magnetic resonance imaging; we will focus on this point in subsequent analyses and investigate the effect factors of image quality or heart size.

## Conclusion

In conclusion, this study showed that semiautomated measurements by the single-beat RT-3DE method had low bias compared with the 2DE Simpson’s method for assessing LA volume and function in AF patients. Compared with 2DE, RT-3DE had the advantage of displaying the 3D anatomy directly. With more experience, RT-3DE may be more acceptable and feasible in clinical practice for AF patients than conventional 2DE.

## Additional files

Additional file 1: Figure S1. The short axis of the left ventricle, apical four-chamber, three-chamber, and short-axis images. (TIFF 689 kb)

Additional file 2: Figure S2. Full volume image of the left atrium from healthy controls (A) and patient cases (B). (ZIP 1549 kb)

Additional file 3: Figure S3. Manual tracking of the left atrial endocardium from healthy controls (A) and patient cases (B). (ZIP 1184 kb)

## Abbreviations

2DE: Two-dimensional echocardiography
AF: Atrial fibrillation
BSA: Body surface area
DCG: Dynamic electrocardiography
ECG: Electrocardiography
EF: Ejection fraction
ILAEDV: Indexed LA end-diastole volume
ILAESV: Indexed LA end- systolic volume
LA: Left atrial
LAEF: LA ejection fraction
RT-3DE: real-time three-dimensional echocardiography
SD: Standard deviation
