# Supplementary figures and images for: Evaluation of left atrial volume and function using single-beat real-time three-dimensional echocardiography in atrial fibrillation patients
Source: BMC Med Imaging. 2017 Jul 21;17:44. doi: 10.1186/s12880-017-0215-7 (PMC5521085; doi:10.1186/s12880-017-0215-7)

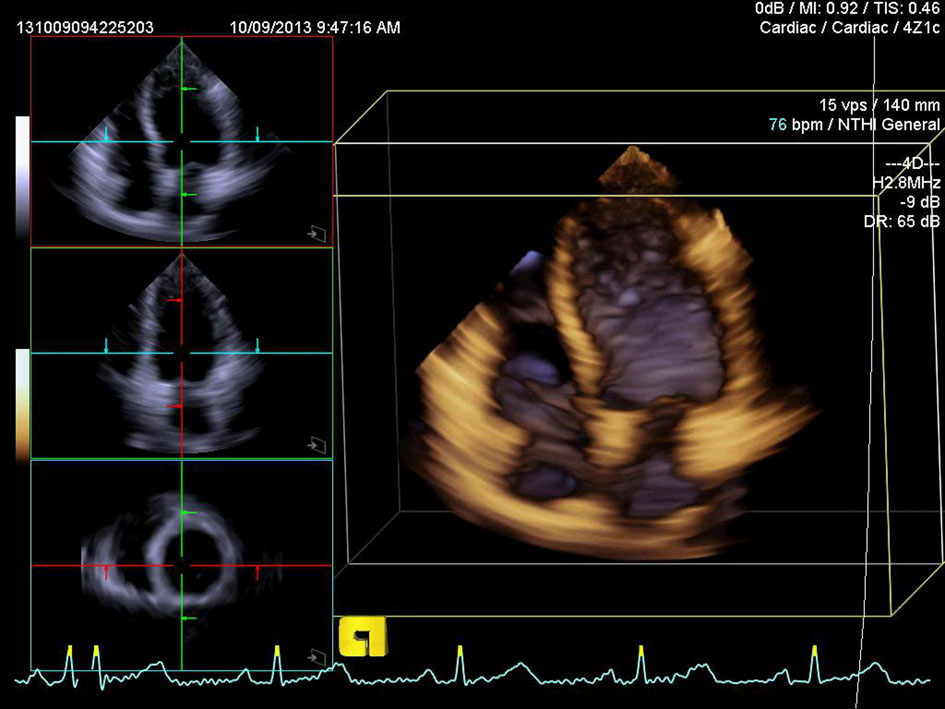

Supplement: Supplementary file 1 — The short axis of the left ventricle, apical four-chamber, three-chamber, and short-axis images. (TIFF 689 kb) [file 12880_2017_215_MOESM1_ESM.tif]

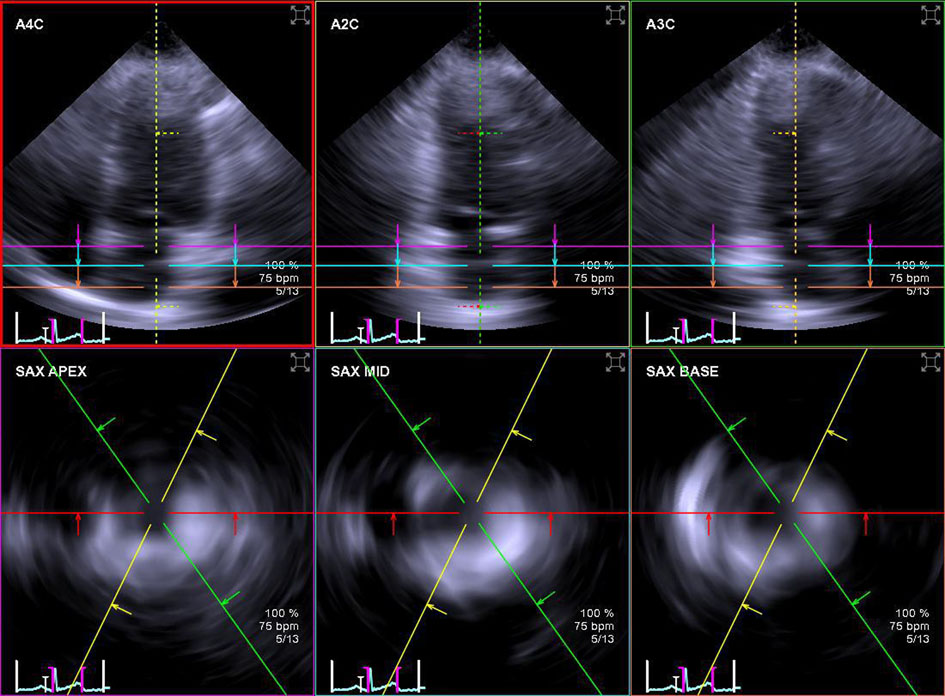

Supplement: Supplementary file 2 — Full volume image of the left atrium from healthy controls (A) and patient cases (B). (ZIP 1549 kb) [file 12880_2017_215_MOESM2_ESM.zip › Supplementary Figure 2AR2.tif]

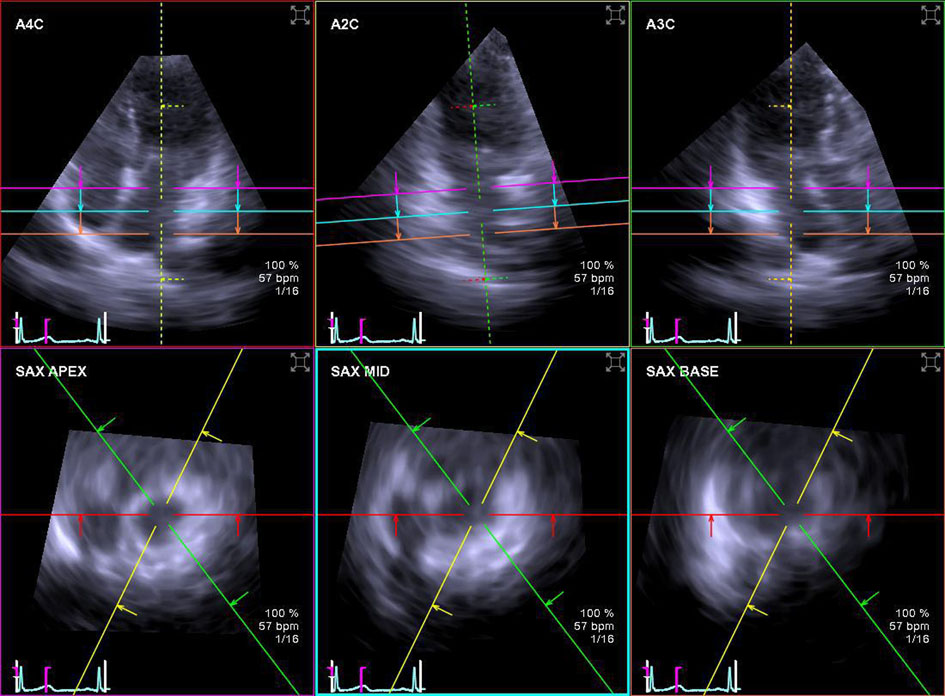

Supplement: Supplementary file 2 — Full volume image of the left atrium from healthy controls (A) and patient cases (B). (ZIP 1549 kb) [file 12880_2017_215_MOESM2_ESM.zip › Supplementary Figure 2BR2.tif]

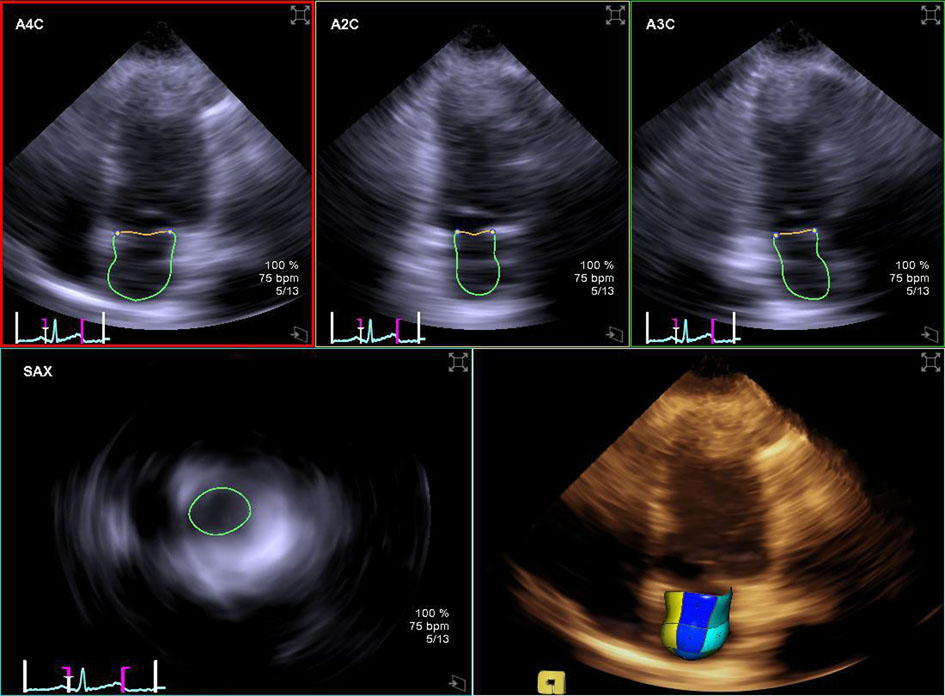

Supplement: Supplementary file 3 — Manual tracking of the left atrial endocardium from healthy controls (A) and patient cases (B). (ZIP 1184 kb) [file 12880_2017_215_MOESM3_ESM.zip › Supplementary Figure 3AR2.tif]

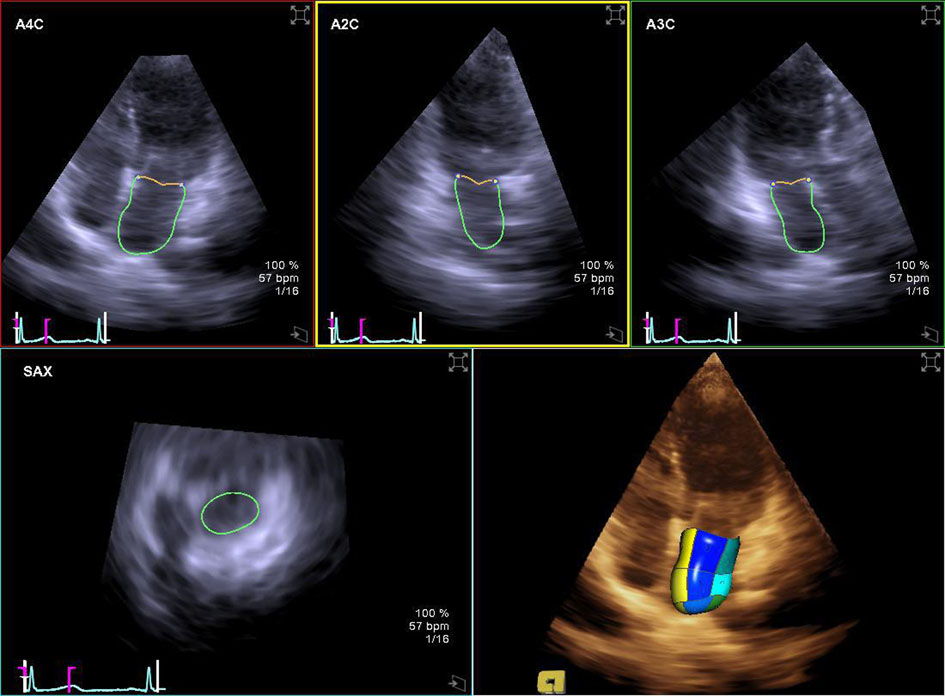

Supplement: Supplementary file 3 — Manual tracking of the left atrial endocardium from healthy controls (A) and patient cases (B). (ZIP 1184 kb) [file 12880_2017_215_MOESM3_ESM.zip › Supplementary Figure 3BR2.tif]
